# Prevalence and Patterns of Non‐Medical Gabapentinoid Use in a General Population Sample: Findings From the Australian National Drug Strategy Household Survey

**DOI:** 10.1111/dar.70165

**Published:** 2026-04-27

**Authors:** Amy G. McNeilage, Ting Xia, Bridin Murnion, Claire E. Ashton‐James, Suzanne Nielsen

**Affiliations:** ^1^ Monash Addiction Research Centre, Eastern Health Clinical School Monash University Melbourne Australia; ^2^ Sydney Medical School, Faculty of Medicine and Health The University of Sydney Sydney Australia; ^3^ School of Clinical Medicine, St Vincent's Healthcare Clinical Campus, Faculty of Medicine and Health University of New South Wales Sydney Australia

**Keywords:** Australia, gabapentin, pregabalin, prescription drug misuse, prevalence

## Abstract

**Introduction:**

Gabapentinoid prescribing and related harms are increasing internationally, yet population‐level evidence on non‐medical use in Australia is lacking. This study estimated the prevalence and correlates of past‐year non‐medical gabapentinoid use and examined temporal trends.

**Methods:**

Data were pooled from the 2016, 2019 and 2022–2023 National Drug Strategy Household Surveys (*N* = 67,103). Analyses applied person‐level survey weights and accounted for the complex design. Weighted descriptive, trend and multinomial logistic regression analyses were conducted, with covariates prespecified using a directed acyclic graph.

**Results:**

Past‐year non‐medical gabapentinoid use among Australians aged ≥ 14 years increased from 0.06% in 2016 to 0.15% in 2022–2023 (Adjusted Wald *F* = 5.86, *p* = 0.016; OR = 1.57, 95% CI 1.09, 2.25). Among those reporting any non‐medical pain medication use, the proportion involving gabapentinoids rose from 1.7% to 9.3%. Individuals reporting non‐medical gabapentinoid use were more likely to report chronic pain (adjusted relative risk ratio [aRRR] = 5.66, 95% CI 3.18, 10.08), high psychological distress (aRRR = 4.26, 95% CI 2.31, 7.84), and socioeconomic disadvantage (aaRRR = 3.45, 95% CI 1.91, 6.27), compared to those reporting no non‐medical pain medication use. Most (71%) also reported non‐medical opioid use.

**Discussion and Conclusions:**

Although non‐medical gabapentinoid use remains uncommon in Australia, its prevalence is increasing. Findings are consistent with non‐medical use occurring in the context of unmet pain and mental health needs rather than predominantly recreational use. Enhanced prescriber awareness, harm reduction initiatives, and expanded access to non‐pharmacological pain and mental health care are needed.

## Summary

- This study provides the first nationally representative estimate of non‐medical gabapentinoid use in Australia.
- Non‐medical gabapentinoid use has increased steadily since 2016.
- Use is strongly associated with chronic pain, high psychological distress and socioeconomic disadvantage.
- Most people reporting non‐medical gabapentinoid use also reported non‐medical prescription opioid use.

## 1 Introduction

Gabapentinoid use is increasing globally [1, 2]. The gabapentinoid drugs—pregabalin and gabapentin—were originally developed for epilepsy but are now mostly prescribed for pain [3]. Evidence supports their efficacy for certain neuropathic pain conditions, such as postherpetic neuralgia and diabetic neuropathy [4, 5]. However, their widespread off‐label use for non‐neuropathic pain [6, 7, 8] is generally not evidence‐based [3]. Healthcare practitioners have attributed overprescribing partly to increased caution around opioids and limited access to non‐pharmacological pain treatments [9].

In the United States (US), gabapentin is prescribed more often than pregabalin [8], largely because pregabalin is federally controlled whereas gabapentin is not [10]. In Australia, both pregabalin and gabapentin are prescription‐only Schedule 4 medicines and are included in real‐time prescription monitoring systems in most jurisdictions. However, pregabalin is subsidised via the Pharmaceutical Benefits Scheme, whereas gabapentin has more limited subsidised indications [7]. Following the introduction of government subsidy in 2013, pregabalin dispensing rose sharply through to 2017 before stabilising [7, 11]. By 2018, Australia recorded the highest rate of pregabalin consumption worldwide [1]. Gabapentin is also used in Australia but at much lower levels [1].

Non‐medical use of gabapentinoids is now well documented internationally [12]. Motivations for such use include psychoactive effects, modifying other drug effects (e.g., potentiating the effects of opioids or offsetting the effects of stimulants), and self‐medication [13]. Such use is associated with increased risk of harm, including central nervous system (CNS) depression and overdose, particularly when combined with opioids or other sedating substances [14]. Australian data suggest the problem is growing: pregabalin misuse‐related ambulance attendances in Victoria increased tenfold between 2012 and 2017 [15], and poisonings and deaths have also risen [16, 17]. In 2023, gabapentinoids were involved in 12.4% of unintentional drug‐induced deaths in Australia, up from 0.1% in 2001, with almost all cases involving other drugs (97%), most commonly opioids and benzodiazepines [18, 19].

It has been estimated that one in seven Australians prescribed pregabalin are at high risk of non‐medical use [16]. Among people who inject drugs, one‐quarter reported pregabalin use in the past 6 months, with 15% using it non‐medically [20]. While such studies demonstrate non‐medical gabapentinoid use in Australia, its prevalence in the general population remains unknown. Understanding the nature and extent of this phenomenon is essential to inform public health responses and policy.

This study uses data from the National Drug Strategy Household Survey (NDSHS) to characterise non‐medical gabapentinoid use in Australia. The primary objective was to estimate past‐year prevalence, while secondary objectives were to describe the characteristics and context of use, identify health and sociodemographic correlates, and assess trends over time.

## 2 Methods

### 2.1 Study Design and Data Source

This cross‐sectional study used data from the 2016, 2019 and 2022–2023 waves of the NDSHS, a nationally representative survey of alcohol, tobacco and other drug use in Australia [21]. These were the first NDSHS waves to include a question on past‐year non‐medical gabapentinoid use. The survey is conducted triennially by the Australian Institute of Health and Welfare (AIHW) using multi‐stage stratified random sampling of private dwellings. The survey uses a multimode design; in each wave the majority of respondents (> 70%) completed a paper form, a small proportion (< 0.5%) completed the survey by telephone interview, and the remainder completed the survey online. Within each selected household, the resident aged ≥ 14 years with the most recent birthday is invited to participate. Survey estimates are weighted to account for selection probabilities and non‐response, and to align the sample with the Australian population. Detailed methodology is published elsewhere [22]. Response rates were 51.1% (2016), 49.0% (2019) and 43.9% (2022–2023).

Ethics approval for data collection was provided by the AIHW Ethics Committee, and secondary analysis was approved by the Monash University Human Research Ethics Committee (Project ID: 45196).

### 2.2 Data Cleaning and Harmonisation

Variables were harmonised across survey waves for comparability (Table S1). The sex/gender measure varied between waves: the 2016 and 2019 surveys asked “What is your sex?”, whereas the 2022–2023 survey used the Australian Bureau of Statistics standard items on sex and gender. For time‐series comparisons, 2016/2019 data were reported by sex and 2022–2023 data by gender, consistent with AIHW guidance [23]. Respondents aged 12–13 years (sampled in 2016 only) were excluded, and several variables were collapsed to ensure stable estimates (Table S2).

### 2.3 Measures

#### 2.3.1 Non‐Medical Gabapentinoid Use (Primary Outcome)

In the NDSHS, non‐medical use is defined as using a drug for recreational purposes to induce or enhance a drug experience, or using a pharmaceutical drug in a way that was not prescribed or recommended. Respondents reporting any non‐medical use of pain medications were asked about past‐year use of specific drugs, including gabapentinoids. Item wording varied slightly across waves: “Gabapentinoids (Neurontin, Lyrica)” in 2016, “Gabapentinoids (e.g., Lyrica)” in 2019, and “Gabapentin or pregabalin (e.g., Lyrica, Neurontin)” in 2022–2023. Because this item was nested within the non‐medical pain medication module, respondents who did not identify gabapentinoids as pain medications were not assessed.

#### 2.3.2 Non‐Medical Opioid Use

Past‐year non‐medical use of codeine, morphine, fentanyl, tramadol, oxycodone and tapentadol (added in 2022–2023) was assessed. In 2016, both prescription and over‐the‐counter (OTC) codeine were listed as separate response options. Because OTC codeine products were rescheduled to prescription‐only in 2018 and were not included in the 2019 or 2022–2023 surveys, 2016 cases reporting only OTC codeine were excluded. Cases reporting only non‐medical use of “other” pain medications were also excluded.

#### 2.3.3 Characteristics of Non‐Medical Pain Medication Use

Respondents who reported any non‐medical use of pain medications were then asked a set of general follow‐up questions (not specific to individual drugs) on recency, frequency, source, setting, concurrent drug use, and difficulty stopping or reducing.

#### 2.3.4 Other Substance Use and Health Variables

Derived variables captured lifetime illicit non‐pharmaceutical drug use, lifetime injecting drug use, and past‐year use of other illicit or prescription substances. Self‐rated general health was assessed with a single item (“In general, would you say your health is…?” response options: excellent, very good, good, fair or poor). Mental health and chronic pain diagnoses were based on past‐year treatment or diagnosis. Current psychological distress was assessed using the 10‐item Kessler Scale [24].

#### 2.3.5 Sociodemographic Variables

Variables included age, sex/gender, relationship status, household size, employment status, education level, personal income, geographic remoteness and socioeconomic status. Socioeconomic status was based on SEIFA Index of Relative Socio‐economic Advantage and Disadvantage quintiles [25]. Geographic remoteness was classified according to Australian Statistical Geography Standard categories [26].

### 2.4 Analytic Groups

Respondents who answered the past‐year pain medication use items (*n =* 65,828) were classified into three mutually exclusive groups:

1. NMGU: past‐year non‐medical gabapentinoid use (primary group of interest).
2. NMOU: past‐year non‐medical opioid use without gabapentinoids.
3. NoNMU: no past‐year non‐medical opioid or gabapentinoid use.

Respondents reporting NMGU were analysed together regardless of opioid use due to small cell sizes. Given the large class imbalance (95% NoNMU), a stratified random 2.5% subsample of NoNMU respondents (*n* = 1602) was drawn for regression analyses. This subsample did not differ significantly from the full NoNMU group on key variables (Table S3). The NMOU group served as a comparator representing non‐medical use of another prescription analgesic class, while the NoNMU group served as a general‐population reference.

### 2.5 Data Analysis

#### 2.5.1 Pooling Survey Waves

Because non‐medical gabapentinoid use was rare (< 1%), data from the three NDSHS waves were pooled to maximise power, following established guidance for analysing rare events in complex surveys [27]. A completed pooling studies critical appraisal checklist is provided in the Supporting Information (Table S4). Each wave used independent random sampling, making respondent overlap negligible. A categorical survey‐wave variable was entered as a fixed effect in all models to adjust for survey‐specific factors. Quality control checks were undertaken by cross‐tabulating key variables against survey wave, and distributions were similar across waves (Table S5).

#### 2.5.2 Survey Weights

All analyses applied AIHW person‐level survey weights, which account for age, sex/gender, household size and geography [22]. To address unequal sample sizes across waves, original weights were rescaled to reflect each wave's proportional contribution to the pooled dataset, following established methods [28]. Each respondent's person‐level weight was multiplied by an adjustment factor equal to that wave's sample size divided by the total pooled sample size (0.349 for 2016, 0.328 for 2019, 0.323 for 2022–2023).

#### 2.5.3 Missing Data

Patterns of missingness were examined using chi‐square tests. Some associations suggested data were not missing completely at random. Overall, missing data were low for variables included in regression models (< 1% for most variables), with higher levels for chronic pain diagnosis (7.2%) and lifetime illicit non‐pharmaceutical drug use (3.0%). Missingness of this magnitude is generally considered acceptable in survey research [29]. To preserve sample size and minimise potential bias, missing values were retained as separate categories.

#### 2.5.4 Descriptive Analyses

Weighted prevalence estimates with 95% confidence intervals (CI) were calculated. Rao‐Scott *χ* ^2^ tests (categorical) and Wald tests (continuous) assessed group differences. Pairwise contrasts were conducted for NMGU versus NMOU and NMGU versus NoNMU using Bonferroni‐adjusted *α* = 0.025. Trends across survey waves were examined using survey‐weighted logistic regression with wave treated as an ordinal predictor. Following AIHW conventions, estimates with a relative standard error of 25%–50% were flagged as cautious and > 50% as unreliable; very small cells (< 5 or base < 30) were suppressed. Descriptive analyses were conducted in IBM SPSS Statistics 31 (IBM Corp).

#### 2.5.5 Regression Analyses

Weighted multinomial logistic regression examined factors associated with NMGU and NMOU (reference: NoNMU). Models incorporated the NDSHS complex design (weights, stratification, clustering). Covariates were prespecified using a directed acyclic graph (Figure S1). Although causal directionality cannot be established in a cross‐sectional design, directed acyclic graphs provide a useful framework for clarifying assumed relationships and identifying a minimally sufficient adjustment set [30]. The final model adjusted for age, sex/gender, chronic pain, psychological distress, socioeconomic status and lifetime illicit non‐pharmaceutical drug use, with survey wave entered as a fixed covariate. Sensitivity analyses used complete‐case data and unadjusted AIHW weights. Regression analyses were conducted in Stata 18 (StataCorp).

## 3 Results

### 3.1 Pooled NDSHS Sample

**FIGURE 1:**
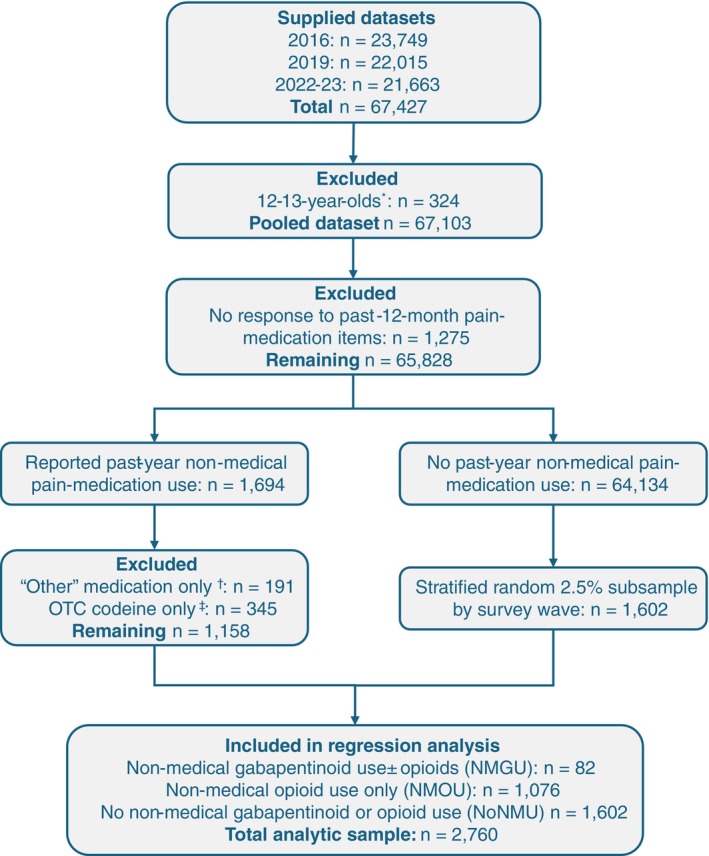
Sample derivation for pooled NDSHS data (2016, 2019, 2022–2023). *12–13‐year‐olds were sampled in 2016 but not in 2019 or 2022–2023; they were excluded to ensure cross‐wave comparability; † Cases reporting non‐medical use limited to “other pain medications” were excluded because specific substances were not identifiable; ‡ In 2016, prescription codeine was retained in the opioid measure; over‐the‐counter (OTC) codeine was excluded to maintain comparability with later waves that captured prescription opioids only.

After excluding 12–13‐year‐olds, the pooled NDSHS sample included 67,103 respondents, weighted to represent approximately 21 million Australians aged ≥ 14 years (Figure 1). Survey‐wave contributions were 23,425 (2016), 22,015 (2019) and 21,663 (2022–2023). Of the pooled sample, 1694 (weighted 2.48%) reported past‐year non‐medical use of pain medication, 64,134 (95.71%) reported no such use, and 1275 (weighted 1.81%) did not answer.

### 3.2 Prevalence of Non‐Medical Gabapentinoid Use

In 2022–2023, 4.93% (95% CI 4.54%, 5.35%) of Australians aged ≥ 14 years reported lifetime non‐medical use of any pain medication, and 2.13% (95% CI 1.90%, 2.39%) reported past‐year use. Among those reporting past‐year use, 9.28% (95% CI 5.98%, 14.13%) reported that this use included gabapentinoids. At the population level, this equated to a weighted prevalence of 0.15% (95% CI 0.10%, 0.24%) for past‐year non‐medical gabapentinoid use, equivalent to 32,709 (95% CI 17,908, 47,510) Australians. For comparison, the weighted prevalence of past‐year non‐medical prescription opioid use was 1.54% (95% CI 1.34%, 1.76%), representing 331,090 (95% CI 284,274, 377,906) Australians.

### 3.3 Trends Over Time

**FIGURE 2:**
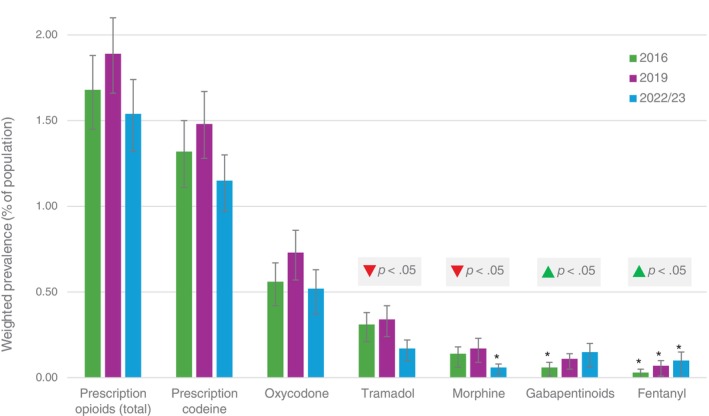
Prevalence of past‐year non‐medical use of prescription pain medications among Australians aged ≥ 14 years, 2016–2022/2023. Error bars represent 95% confidence intervals. *Relative standard error = 25%–50%, interpret with caution. ▲ or ▼ indicates a significant linear trend across survey years; no significant trend was detected for other drug categories.

Past‐year non‐medical gabapentinoid use increased steadily from 0.06% (95% CI 0.03%, 0.11%; relative standard error = 34%) in 2016 to 0.11% (95% CI 0.08%, 0.17%) in 2019 and 0.15% (95% CI 0.10%, 0.24%) in 2022–2023 (Figure 2). Survey‐weighted logistic regression confirmed a significant upward trend (Adjusted Wald *F* = 5.86, *p* = 0.016) with each successive survey wave associated with 1.57 times higher odds (95% CI 1.09, 2.25) of past‐year non‐medical gabapentinoid use. In contrast, the prevalence of past‐year non‐medical prescription opioid use was 1.68% (95% CI 1.48%, 1.91%) in 2016, 1.89% (95% CI 1.68%, 2.12%) in 2019, and 1.54% (95% CI 1.34%, 1.76%) in 2022–2023, with no evidence of a linear trend (Adjusted Wald *F* = 0.91, *p* = 0.340).

Over the same period, lifetime non‐medical use of pain medication (including OTC codeine) fell markedly from 9.59% (95% CI 9.13%, 10.07%) in 2016 to 8.24% (95% CI 7.80%, 8.71%) and 4.93% (95% CI 4.54%, 5.35%) in 2022–2023. Survey‐weighted logistic regression confirmed a significant downward trend (Adjusted Wald *F* = 204.01, *p* < 0.001; odds ratio = 0.71, 95% CI 0.68, 0.75). Past‐year non‐medical use of pain medication (including OTC codeine) also declined, from 3.53% (95% CI 3.25%, 3.84%) in 2016 to 2.62% (95% CI 2.38%, 2.89%) in 2019 and 2.13% (95% CI 1.90%, 2.39%) in 2022–2023 (Adjusted Wald *F* = 51.17, *p* < 0.001; odds ratio = 0.77, 95% CI 0.72, 0.83). This downward trend was driven by reductions in non‐medical use of OTC codeine following its rescheduling in 2018 and is therefore not directly comparable with the prescription opioid estimates shown in Figure 2.

Among respondents reporting any past‐year non‐medical pain medication use, the proportion using gabapentinoids rose sharply, from 1.73% (95% CI 0.89%, 3.34%; relative standard error = 35%) in 2016 to 4.67% (95% CI 3.09%, 7.00%) in 2019 and 9.28% (95% CI 5.98%, 14.13%) in 2022–2023. Survey‐weighted logistic regression confirmed a significant upward trend (Adjusted Wald *F* = 19.35, *p* < 0.001), with each successive survey wave associated with more than double the odds of gabapentinoid use (odds ratio = 2.36, 95% CI 1.61, 3.46).

### 3.4 Characteristics of Analytic Sample

A total of 2760 respondents were included in regression analyses (Figure 1 shows the flow of respondents from the pooled NDSHS sample to the analytic sample). Of these, 82 reported past‐year non‐medical gabapentinoid use (NMGU), 1076 reported past‐year non‐medical opioid use without gabapentinoids (NMOU), and 1602 reported no past‐year non‐medical use of opioids or gabapentinoids (NoNMU). Within the NMGU group, 70.75% (95% CI 56.40%, 81.88%) also reported non‐medical prescription opioid use, most commonly codeine (46.46%, 95% CI 33.29%, 60.15%), oxycodone (39.93%, 95% CI 27.15%, 54.24%), and tramadol (32.09%, 95% CI 21.37%, 45.12%).

Weighted sociodemographic and health characteristics are presented in Table 1. There were no significant differences in age or sex/gender between the NMGU group and either comparator group (all *p* > 0.025). Compared with both NMOU and NoNMU, individuals reporting NMGU were more likely to live alone, reside in socioeconomically disadvantaged areas, and report poor general health, high/very high psychological distress, chronic pain and a mental health diagnosis (all *p* < 0.025).

**TABLE 1:** Weighted sociodemographic and health characteristics by analytic group: Past‐year non‐medical gabapentinoid use (NMGU), non‐medical opioid use without gabapentinoids (NMOU) and no non‐medical use of either (NoNMU).

Characteristic	NMGU (*n* = 82)	NMOU (*n* = 1076)	NoNMU (*n* = 1602)	*p* ^a^	*p* ^b^
Age, mean (SE)	46.2 (2.89)	42.6 (0.68)	44.5 (0.62)	0.229	0.565
Male, %	54.9	54.8	50.6	0.985	0.565
Partnered, %	32.2	47.1	59.7	0.033	< 0.001
Living alone, %	23.5	11.6	8.6	0.002	< 0.001
Above high school education^e^, %	57.5	56.4	61.2	0.889	0.635
Currently employed^e^, %	54.0	68.8	64.1	0.038	0.167
Personal income ≥ $65,000^f^, %	26.2	37.0	37.0	0.156	0.154
Disadvantaged socioeconomic status, %	68.2	43.0	37.9	< 0.001	< 0.001
Regional, remote, very remote area, %	39.7	30.7	27.7	0.211	0.084
Poor general health, %	31.3	18.5	12.3	0.016	< 0.001
High/very high distress, %	52.4	33.7	14.4	0.008	< 0.001
Chronic pain diagnosis^e^, %	47.5	19.7	9.8	< 0.001	< 0.001
Mental health diagnosis^e^, %	52.4	34.2	16.3	0.015	< 0.001
Lifetime illicit non‐pharmaceutical drug use, %	69.6	71.4	38.7	0.804	< 0.001
Lifetime injecting drug use, %	19.3	12.1	1.6	0.161	< 0.001
Past‐year illicit drug use					
Marijuana/cannabis, %	48.4	42.3	10.6	0.421	< 0.001
Methamphetamine/amphetamine, %	23.8^c^	15.7	1.3^c^	0.188	< 0.001
Heroin, %	7.8^c^	3.6	0	0.117	< 0.001
Cocaine, %	16.9^c^	25.1	3.6	0.207	< 0.001
Ecstasy, %	19.0^c^	17.5	2.3	0.798	< 0.001
Hallucinogens, %	17.3^c^	11.6	1.4^c^	0.250	< 0.001
Ketamine, %	9.9^c^	8.0	0.5^c^	0.683	< 0.001
GHB/GBL/1,4B‐D, %	4.4^d^	2.4^c^	0	0.512	< 0.001
Inhalants, %	10.3^c^	7.6	0.8^c^	0.549	< 0.001
Past‐year non‐medical drug use					
Pharmaceutical opioids, %	70.7	100	0	< 0.001	< 0.001
Tranquilisers/sleeping pills, %	34.6	22.5	0.9	0.060	< 0.001
Pharmaceutical stimulants, %	36.2^c^	14.4	2.2^c^	0.016	< 0.001

*Note:* Percentages are weighted and calculated among respondents with non‐missing data (missing responses excluded from denominators). All variables are binary.

^a^ NMOU vs. NMGU.

^b^ NoNMU vs. NMGU (survey‐weighted tests, significance assessed at Bonferroni‐adjusted *α* = 0.025 due to multiple comparisons).

^c^ Relative standard error (RSE) 25%–50%, interpret with caution.

^d^ RSE 51%–90%, estimate considered unreliable.

^e^ Moderate missingness (5%–10%) for this variable.

^f^ Personal income had high missingness (21%), consistent with common nonresponse patterns in survey data.

### 3.5 Characteristics and Context of Non‐Medical Pain Medication Use

Table 2 summarises the characteristics and context of non‐medical pain medication use (not specific to gabapentinoids or opioids), among respondents reporting NMGU or NMOU. Between‐group comparisons were not performed due to small, unweighted cell counts. Among NMGU respondents, onset typically occurred in early adulthood (mean = 23.7 years), and patterns of use were frequent and persistent, with approximately one‐quarter reporting daily use and half indicating difficulty stopping or reducing use in the past year. Prescriptions and friends were the most common sources, use typically occurred at home, and concurrent use with alcohol, tobacco, cannabis and other drugs was common.

**TABLE 2:** Characteristics and context of non‐medical pain medication use by analytic group: past‐year non‐medical gabapentinoid use (NMGU) and non‐medical opioid use without gabapentinoids (NMOU).

Characteristic	NMGU (*n* = 82)		NMOU (*n* = 1076)	
	Weighted %	95% CI	Weighted %	95% CI
Age at first non‐medical use, mean	23.7	(19.8, 27.6)	21.4	(20.5, 22.2)
Past‐week use	32.2	(21.0, 45.9)	18.8	(16.0, 21.9)
Past‐month use	52.1	(38.2, 65.6)	36.6	(33.1, 40.3)
Difficulty reducing (past 12 months)	51.3	(37.3, 65.0)	45.0	(41.2, 48.9)
Use frequency (past 12 months)				
Every day	24.4	(14.8, 37.6)	6.6	(5.1, 8.6)
Once a week or more	21.2	(13.0, 32.6)	14.4	(11.7, 17.6)
About once a month	10.6^a^	(4.1, 24.8)	13.4	(11.1, 16.1)
Every few months	14.4^a^	(6.9, 27.6)	22.2	(19.2, 25.4)
Once or twice a year	25.9^a^	(14.3, 42.2)	35.4	(31.9, 39.0)
Not answered	3.5^a^	(1.1, 10.3)	8.0	(6.4, 10.0)
Initial source				
Prescription for medical condition	38.0	(25.7, 51.9)	41.4	(37.5, 45.4)
Friend	29.8^a^	(16.5, 47.5)	20.1	(16.9, 23.9)
Relative	16.3^a^	(7.9, 30.8)	9.1	(7.0, 11.8)
‘Doctor shopping’ or forged script	8.1^a^	(3.6, 17.2)	7.3	(5.6, 9.6)
Other	7.9^a^	(3.0, 19.0)	22.0	(19.0, 25.3)
Usual source				
Prescription for a medical condition	31.7	(19.8, 46.5)	37.7	(33.9, 41.5)
Friend	25.1^a^	(12.8, 43.3)	19.5	(16.2, 23.3)
Relative	15.3^a^	(6.5, 31.9)	9.1	(6.8, 11.9)
‘Doctor shopping’ or forged script	10.6^a^	(4.7, 21.9)	7.2	(5.4, 9.5)
Other	17.4^a^	(6.9, 37.4)	26.5	(23.2, 30.2)
Usual location of use^b^				
Own or partner's home	81.4	(69.5, 89.3)	76.6	(73.1, 79.7)
Friend's house	19.6^a^	(10.1, 34.6)	12.2	(9.6, 15.1)
In public places (e.g., park, beach)	10.3^a^	(4.0, 23.9)	2.2^a^	(1.2, 4.0)
Concomitant drugs (on at least one occasion)^b^				
Alcohol	45.9	(32.2, 60.2)	42.1	(38.4, 45.9)
Tobacco	37.8	(25.2, 52.5)	28.3	(24.9, 32.0)
Marijuana/cannabis	34.8	(22.5, 49.6)	22.9	(19.6, 26.6)
Tranquilisers or sleeping pills	26.4	(16.2, 40.0)	6.5	(5.0, 8.5)
Methamphetamine or amphetamine	17.0^a^	(8.3, 31.7)	7.7	(5.7, 10.4)
Cocaine/crack	10.8^a^	(5.2, 21.1)	6.8	(5.2, 9.0)
Ecstasy	10.7^a^	(5.1, 21.3)	5.1	(3.8, 7.0)
Heroin	5.2^a^	(2.2, 12.4)	2.3	(1.5, 3.6)
None	23.3	(14.4, 35.2)	36.0	(32.5, 39.7)

*Note:* Survey questions referred to non‐medical use of pain medications in general, not specifically to gabapentinoids or opioids. Categories with fewer than five unweighted responses are not reported, in accordance with AIHW reporting standards.

Abbreviation: CI, confidence interval.

^a^ Relative standard error of 25%–50%, interpret with caution.

^b^ Multiple responses permitted.

### 3.6 Factors Associated With Non‐Medical Gabapentinoid Use

Survey‐weighted multinomial logistic regression (Table 3) showed that chronic pain and psychological distress were associated with increased relative risk of being in both NMGU and NMOU groups versus NoNMU, with larger effects for NMGU. Lifetime illicit non‐pharmaceutical drug use was likewise associated with higher relative risk of being in either group versus NoNMU, although the association was stronger for NMOU. Socioeconomic disadvantage was significant only for NMGU. Neither age nor sex/gender was associated with NMGU or NMOU versus NoNMU. Sensitivity analyses using complete‐case data (Table S6) and unadjusted survey weights (Table S7) produced consistent results.

**TABLE 3:** Multinomial logistic regression of factors associated with past‐year non‐medical gabapentinoid use (NMGU) and non‐medical opioid use without gabapentinoids (NMOU), compared with no non‐medical use of either (NoNMU).

Variable	NMGU (*n* = 82)		NMOU (*n* = 1076)	
	RRR (95% CI)	*p*	RRR (95% CI)	*p*
Age (continuous)	1.01 (0.99–1.03)	0.399	1.00 (1.00–1.01)	0.302
Female sex/gender	1.00 (0.55–1.81)	0.998	0.90 (0.74–1.10)	0.295
Chronic pain diagnosis	5.66 (3.18–10.08)	< 0.001	1.84 (1.38–2.46)	< 0.001
High/very high psychological distress	4.26 (2.31–7.84)	< 0.001	2.38 (1.84–3.09)	< 0.001
Disadvantaged socioeconomic status	3.45 (1.91–6.27)	< 0.001	1.22 (0.99–1.51)	0.060
Lifetime illicit non‐pharmaceutical drug use	3.01 (1.33–6.82)	0.008	3.53 (2.84–4.39)	< 0.001
2019 survey wave	2.01 (0.87–4.66)	0.103	1.02 (0.80–1.29)	0.903
2022–2023 survey wave	2.37 (1.00–5.61)	0.050	0.75 (0.58–0.97)	0.026

*Note:* Reference group = NoNMU. CI, confidence interval; RRR, relative risk ratio. Analyses account for the National Drug Strategy Household Survey complex survey design. Models adjusted for age, sex/gender, chronic pain, psychological distress, socioeconomic status, lifetime illicit non‐pharmaceutical drug use, and survey wave. Reference categories: male sex/gender, no chronic pain diagnosis, low/moderate psychological distress, disadvantaged socioeconomic status, no lifetime illicit non‐pharmaceutical drug use, and 2016 survey wave. Missing data were retained as a separate category for chronic pain, psychological distress, and lifetime illicit non‐pharmaceutical drug use (results not shown).

## 4 Discussion

This study provides the first nationally representative prevalence estimate of non‐medical gabapentinoid use in Australia. Although non‐medical use remains uncommon, affecting an estimated 0.15% of Australians in 2022–2023, the prevalence has risen steadily since 2016. Among those reporting past‐year non‐medical pain medication use, the proportion using gabapentinoids increased from 1.7% to 9.3%, indicating a substantial shift within this group.

### 4.1 Prevalence and Trends

International surveys report past‐year non‐medical gabapentinoid use ranging from 0.3% to 1.7% in Europe and the US [31, 32], and lifetime prevalence up to 6.6% in the US [33], and between 0.5% and 1.1% in the United Kingdom [34]. Although rates appear lower in Australia, differences in definitions and sampling complicate comparisons.

Despite its relative rarity, non‐medical gabapentinoid use in Australia has grown consistently, with each successive NDSHS wave associated with 1.6 times higher odds of past‐year use. This contrasts with stable or declining non‐medical opioid use. Notably, these increases have occurred despite relatively stable prescribing rates. Pharmaceutical Benefits Scheme data show the number of patients dispensed gabapentinoids fell slightly from approximately 639,000 in 2016–2017 to 618,000 in 2019–2020 and 602,000 in 2022–2023 [35]. The rise in non‐medical use therefore likely reflects behavioural and contextual factors rather than availability.

The trend coincides with tightening regulation of opioids, including the 2018 rescheduling of codeine to prescription‐only status, which substantially reduced overall non‐medical pain medication use. This raises the possibility of a substitution effect, with some individuals—particularly those with chronic pain—shifting toward gabapentinoids amid constrained opioid access. Differences in availability and cost may also contribute to this pattern. Gabapentinoids are relatively inexpensive under the Pharmaceutical Benefits Scheme (maximum co‐payment $25, or $7.70 for concession card holders) and can be dispensed in larger quantities with repeats (e.g., 56 capsules with up to five repeats), whereas opioid prescribing has declined in recent years and is subject to tighter restrictions on pack sizes and repeats [35, 36]. International qualitative evidence has documented gabapentinoids being used non‐medically to substitute for opioids or to manage opioid withdrawal, and that they may be perceived as more accessible, less risky to obtain, and less expensive than opioids [9, 13].

Although non‐medical opioid use remains roughly 10 times more prevalent, opioids are prescribed to a much larger patient base (around five times as many) and had greater population exposure during their period of OTC availability [35]. When considered relative to patient exposure, non‐medical gabapentinoid use appears less common but its consistent upward trajectory warrants attention.

### 4.2 Sociodemographic and Health Correlates

Consistent with prior research [31, 37, 38, 39], non‐medical gabapentinoid use was strongly associated with chronic pain, high psychological distress, prior illicit drug use, and socioeconomic disadvantage, suggesting a population with complex health and social vulnerabilities. These factors (with the exception of prior illicit drug use) were more strongly associated with non‐medical gabapentinoid than opioid use, with relative risk ratios approximately three times higher for chronic pain and socioeconomic disadvantage, and nearly double for psychological distress. However, it should be noted that most individuals reporting non‐medical gabapentinoid use also reported non‐medical opioid use, suggesting that some associations may reflect polysubstance‐related vulnerabilities rather than those specific to gabapentinoids.

Nevertheless, the observed patterns suggest that non‐medical gabapentinoid use in Australia may occur within a context of self‐medication and unmet health needs. Qualitative evidence supports this interpretation with individuals reporting non‐medical use of gabapentinoids to cope with chronic pain and mental health challenges [13, 40]. The association with socioeconomic disadvantage may also reflect structural barriers to comprehensive pain and mental health care [41, 42], prompting reliance on pharmacological coping strategies.

### 4.3 Sources and Context of Non‐Medical Use

Pain medications used non‐medically were most often obtained via prescriptions or friends, suggesting access largely through medical or social channels rather than organised diversion. Furthermore, use typically occurred in the home rather than social settings, consistent with self‐medication behaviours. Although these questions referred to pain medications collectively, limiting drug‐specific inference, findings highlight prescribers and pharmacies as potential intervention points to reduce diversion through better storage and patient education around medication sharing.

High rates of concomitant use with alcohol, cannabis, and other sedatives are concerning, given the well‐documented risks of CNS depression and overdose [14, 43]. Notably, more than two‐thirds of people reporting non‐medical gabapentinoid use also reported non‐medical opioid use. In addition, concomitant use of tranquilisers or sleeping pills was relatively common among those reporting non‐medical gabapentinoid use (26.4%) and substantially higher than among those reporting non‐medical opioid use without gabapentinoids (6.5%). These patterns may compound overdose risk, particularly as those in the gabapentinoid group were more likely to live alone and typically used at home.

### 4.4 Implications for Practice, Policy, and Research

The increasing non‐medical use of gabapentinoids, particularly among people with pain, psychological distress, and socioeconomic disadvantage, calls for proactive clinical and public health responses. Key priorities should include improving prescriber education on the limited efficacy for non‐neuropathic pain [44], the potential for dependence [45], and the risks associated with concomitant use with CNS depressants [14, 43]; implementing harm reduction approaches addressing polysubstance use, including targeted messaging on the increased risk of respiratory depression and overdose when combining gabapentinoids with other sedating medications such as opioids and benzodiazepines; and expanding access to non‐pharmacological pain and mental health care. Given that about half of those reporting non‐medical gabapentinoid use indicated difficulty stopping or reducing pain medication, additional treatment and support for dependence and withdrawal should also be prioritised. Policy measures must remain balanced to avoid unintended shifts toward other substances or barriers for patients with therapeutic need.

Further research should clarify trajectories from prescribed to non‐medical use and identify predictors of escalation or dependence. Longitudinal and data‐linkage studies could illuminate pathways and harms, while qualitative work with prescribers and patients can explore clinical reasoning, system pressures and lived experiences. Evaluating the effects of regulatory and prescribing changes will be crucial to guide future policy.

### 4.5 Limitations

Several factors may have contributed to underestimation of non‐medical gabapentinoid use. The relevant item was administered only to respondents reporting non‐medical use of “pain‐killers/pain‐relievers and opioids,” a gatekeeping design that likely excluded individuals who do not conceptualise gabapentinoids as analgesics (e.g., those using them primarily for sleep, anxiety, recreationally, or to modify the effects of other drugs). This undercount is unlikely to be random and may have introduced differential misclassification, limiting the generalisability of prevalence estimates. In addition, the example drugs listed in the question included only opioids, which may have further reduced reporting.

Because the survey samples only private households, high‐risk or marginalised populations—such as individuals living in institutional settings or experiencing homelessness—are underrepresented, further limiting generalisability. As with all self‐report surveys, social desirability and recall biases may have also contributed to underreporting. Finally, the small number of respondents reporting non‐medical gabapentinoid use (*n* = 82) limited statistical power and precision, and minor wording differences across survey waves may also have affected recognition and reporting.

## 5 Conclusion

Non‐medical use of gabapentinoids remains uncommon in Australia but is increasing, particularly among people with chronic pain, psychological distress and socioeconomic disadvantage, noting that prevalence is likely underestimated due to survey design constraints. These findings are consistent with non‐medical use occurring alongside unmet health and social needs. Careful prescribing, harm reduction focused on overdose prevention, and expanded access to non‐pharmacological pain and mental health care are critical to mitigating harms.

## Author Contributions

Each author certifies that their contribution to this work meets the standards of the International Committee of Medical Journal Editors.

## Funding

Amy G. McNeilage receives an Australian Government Research Training Program Scholarship and a supplementary PhD scholarship from the Pain Foundation. Suzanne Nielsen is the recipient of an NHMRC Investigator Grant Fellowship [grant number 2025894].

## Conflicts of Interest

The authors declare no conflicts of interest.

## Supporting information


**Table S1:** Variable harmonisation across survey waves.
**Table S2:** Variable coding and recategorisation.
**Table S3:** Comparison of key sociodemographic and health characteristics between the NoNMU subsample and the remainder of the full NoNMU sample.
**Table S4:** Completed pooling studies critical appraisal checklist.
**Table S5:** Distribution of key variables by survey wave (total sample, weighted %).
**Table S6:** Excluding missing cases.
**Table S7:** Using original unadjusted survey weight.
**Figure S1:** Directed acyclic graph and covariate adjustment strategy.

## Data Availability

The data that support the findings of this study are available from the Australian Institute of Health and Welfare. Restrictions apply to the availability of these data, which were used under license for this study. Data are available from https://dataverse.ada.edu.au/dataverse/ndshs with the permission of the Australian Institute of Health and Welfare.
