# Supplementary material for: Prevalence and Patterns of Non‐Medical Gabapentinoid Use in a General Population Sample: Findings From the Australian National Drug Strategy Household Survey
Source: Drug Alcohol Rev. 2026 Apr 27;45:e70165. doi: 10.1111/dar.70165 (PMC13112056; doi:10.1111/dar.70165)
Supplement: Supplementary file 1 — Table S1: Variable harmonisation across survey waves. Table S2: Variable coding and recategorisation. Table S3: Comparison of key sociodemographic and health characteristics between the NoNMU subsample and the remainder of the full NoNMU sample. Table S4: Completed pooling studies critical appraisal checklist. Table S5: Distribution of key variables by survey wave (total sample, weighted %). Table S6: Excluding missing cases. Table S7: Using original unadjusted survey weight. Figure S1: Directed acyclic graph and covariate adjustment strategy. [file DAR-45-0-s001.docx]

**Table S1. Variable harmonisation across survey waves**

| **NDSHS variable** | **Variable meaning** | **2016 categories** | **2019 categories** | **2022-23 categories** | **Harmonisation approach** |
| --- | --- | --- | --- | --- | --- |
| **STRATA** | Strata | 1- Sydney  2- Rest NSW  3- Melbourne  4- Rest VIC  5- Brisbane  6- Rest QLD  7- Perth  8- Rest WA  9- Adelaide  10- Rest SA  11- Hobart  12- Rest TAS  13- ACT  14- Darwin  15- Rest NT | 1- Sydney  2- Rest NSW  3- Melbourne  4- Rest VIC  5- Brisbane  6- Rest QLD  7- Adelaide  8- Rest SA  9- Perth  10- Rest WA  11- Hobart  12- Rest TAS  13- Darwin  14- Rest NT  15- ACT | 1- Sydney  2- Rest NSW  3- Melbourne  4- Rest VIC  5- Brisbane  6- Rest QLD  7- Adelaide  8- Rest SA  9- Perth  10- Rest WA  11- Hobart  12- Rest TAS  13- Darwin  14- Rest NT  15- ACT | 2016 recoded to align with 2019/2022–23 structure (Perth ↔ Adelaide; Rest WA ↔ Rest SA; ACT/Darwin/Rest NT rotated). |
| **SA1_D** | Cluster variable | Deidentified numeric codes | Deidentified numeric codes | Deidentified numeric codes | Values are de-identified and have no interpretable categories. No harmonisation required. |
| **SA2_D** | Cluster variable | Deidentified numeric codes | Deidentified numeric codes | Deidentified numeric codes | Values are de-identified and have no interpretable categories. No harmonisation required. |
| **Weight_7** | Person weight | Continuous variable | Continuous variable | Continuous variable | No harmonisation required. |
| **B1** | General health | 1- Excellent  2- Very good  3- Good  4- Fair  5- Poor  -2- Not answered | Same | Same | No change required. |
| **F1** | Lifetime use pain medication | 1- Yes  2- No  -2- Not answered | Same | Same | No change required. |
| **F2** | Lifetime non-medical use pain medication | 1- Yes  2- No  -2- Not answered | Same | Same | No change required. |
| **F3N** | Age when first used pain medication non-medically | Numeric age | Numeric age | Numeric age | No harmonisation required. |
| **F4A** | Past-year non-medical use pain medication | 1- Yes  2- No  -2- Not answered | Same | Same | No change required. |
| **F4B** | Which pain medication used non-medically in the past year | 00- OTC codeine  01- Codeine  02- Morphine  03- Fentanyl  04- Tramadol  05- Oxycodone  07-Gabapentinoids  10- Other | 01- Codeine  02- Morphine  03- Fentanyl  04- Tramadol  05- Oxycodone  07-Gabapentinoids  10- Other | 01- Codeine  02- Morphine  03- Fentanyl  04- Tramadol  05- Oxycodone  06- Tapentadol  07-Gabapentinoids  10- Other | The 2016 category 00 = OTC codeine was dropped, and 06 = Tapentadol (introduced in 2022–23) was added. Codes retained and aligned across waves for comparability. |
| **F5** | Difficulty stopping reducing | 1- Yes (last 12 mo)  2- No  -2- Not answered | 1- Yes (last 3 mo)  2- Yes (last 12 mo)  3- No  -2- Not answered | 1- Yes (last 3 mo)  2- No  3- Yes (last 12 mo)  -2- Not answered | Recoded into three categories: **1 = Yes (past 12 months),** including both 3- and 12-month responses; **2 = No**; -2 **= Not answered**. |
| **F6** | Past month use | 1- Yes  2- No  -2- Not answered | Same | Same | No change required. |
| **F7** | Past week use | 1- Yes  2- No  -2- Not answered | Same | Same | No change required. |
| **F8** | Frequency of use | 1- Every day  2- Once a week or more  3- About once a month  4- Every few months  5- Once or twice a year  -2- Not answered | Same | Same | No change required. |
| **F9A** | Source first obtainment | 1- Shop  2- Friend  3- Relative  4- Partner  5- Dealer  6- Doctor shopping  7- Prescription  8- Internet  9- Stole  10- Other  -2- Not answered | 1- Friend  2- Relative  3- Partner  4- Dealer  5- Doctor shopping  6- Prescription  7- Internet  8- Stole  9- Other  -2- Not answered | 2- Friend  3- Relative  4- Partner  5- Dealer  6- Doctor shopping  7- Prescription  8- Internet  11- Stole  12- Other  -2- Not answered | Harmonised into consistent source categories: Friend, Relative, Partner, Dealer, Doctor shopping, Prescription, Internet, Stole, Other. |
| **F9B** | Source usual obtainment | 1- Shop  2- Friend  3- Relative  4- Partner  5- Dealer  6- Doctor shopping  7- Prescription  8- Internet  9- Stole  10- Other  -2- Not answered | 1- Friend  2- Relative  3- Partner  4- Dealer  5- Doctor shopping  6- Prescription  7- Internet  8- Stole  9- Other  -2- Not answered | 2- Friend  3- Relative  4- Partner  5- Dealer  6- Doctor shopping  7- Prescription  8- Internet  11- Stole  12- Other  -2- Not answered | Harmonised into consistent source categories: Friend, Relative, Partner, Dealer, Doctor shopping, Prescription, Internet, Stole, Other. |
| **F10** | Location of use | 01- Own/ Partner home  02- Friend home 03- Party  04- Rave  05- Restaurant /café  06- Licensed venue  07- School / TAFE/ university  08- Workplace 09 - Public place 10- Car  11- Other | Same | Same | No change required. |
| **F11** | Concomitant substances used | 01- Alcohol  02- Tobacco  03- Tranquillisers  04- Steroids  05- Inhalants  06- Marijuana  07- Hallucinogens 08- Methamphetamine 09- Heroin  10- Cocaine  11- Ecstasy  12- GHB  13- Ketamine  14- Kava  15- Other  16- None | Same | Same | No change required. |
| **Gender (2022-23)**  **Sex (2016, 2019)** | Gender / Sex | 1- Males  2- Females | 1- Males  2- Females | -1- Missing  1- Man on Male  2- Woman or Female  3- Non-binary, other, not answered | Harmonised binary variable (1 = Male/Man; 2 = Female/Woman). Non-binary category coded ‘missing/other’. |
| **CURF_Age** | Age | Continuous variable | Continuous variable | Continuous variable | No harmonisation required. |
| **MaritalStatus** | Current marital status | -1- Missing  1- Never married  2- Divorced/ separated/ widowed  3- Married/ de facto | Same | Same | No change required. |
| **EmploymentALL** | All forms of employment | -1- Missing  1- Not in labour force  2- Unemployed/ looking for work  3- Currently employed | Same | Same | No change required. |
| **HIGHQUAL, ZZ9 (2016) / EduStatus (2019, 2022023)** | Education status | HIGHQUAL:  -2- Missing  1- Certificate I or II 2- Certificate III or IV  3- Associate Diploma  4- Undergraduate Diploma  5- Bachelor Degree 6- Master / Postgraduate / Doctorate  ZZ9 (Highest year of school completed):  -2- Not answered  1- Year 6 or below  3- Year 7 or equivalent  4- Year 8 or equivalent  5- Year 9 or equivalent  6- Year 10 or equivalent  7- Year 11 or equivalent  8- Year 13 or equivalent | EduStatus: 1- Year 11 or less (incl. Certificate I–II)  2- Completed Year 12  3- Certificate III or IV 4- Diploma  5- Bachelor degree or higher | EduStatus: 1- Year 11 or less (incl. Certificate I–II)  2- Completed Year 12  3- Certificate III or IV 4- Diploma  5- Bachelor degree or higher | 2016 HIGHQUAL and ZZ9 combined to derive a new EduStatus variable consistent with 2019/2022–23 coding. Missing and Not answered values coded as system missing. |
| **RcntMari, RcntEcst, RcntAmph, RcntCoca, RcntInha, RcntHall, RcntHero, RcntKeta, RcntGHB, RcntTran, RcntSter, RcntStim** | Past-year use of substances | -1- Missing  1- Yes  2-No | Same | Same | No change required. |
| **EverInj** | Lifetime injecting drug use | -1- Missing  1- Yes  2-No | Same | Same | No change required. |
| **K10 rank** | Psychological distress | 0- Missing  1- Low  2- Moderate  3- High  4- Very high | Same | Same | No change required. |
| **SEIFA** | Index of relative socioeconomic advantage and disadvantage | 1- Lowest  2- 2  3- 3  4- 4  5- Highest | Same | Same | No change required. |
| **NumberPpl** | Household size | Numeric count | Same | Same | No change required. |
| **PersIncome** | Personal income | -2- Not answered  -1- Don’t know  1- $156,000 or more ($3,000 or more/week)  2- $104,000 - $155,999 ($2,000 - $2,999/week)  3- $91,000 - $103,999 ($1,750 - $1,999/week)  4- $78,000 - $90,999 ($1,500 - $1,749/week)  5- $65,000 – $77,999 ($1,250 – $1,499/week)  6- $52,000 – $64,999 ($1,000 – $1,249/week)  7- $41,600 – $51,999 ($800 – $999/week)  8- $33,800 – $41,599 ($650 – $799/week)  9- $26,000 – $33,799 ($500 – $649/week)  10- $20,800 – $25,999 ($400 – $499/week)  11- $15,600 – $20,799 ($300 – $399/week)  12- $7,800 – $15,599 ($150 – $299/week  13- $1 – $7,799 ($1 – $149/week)  14- Nil or negative income  16- Prefer not to say | Same | Same | No change required. |
| **ASGS3** | Remoteness areas for QLD, SA, WA, NT | 1- Major cities  2- Inner regional  3- Outer regional/ remote/ very remote | Same | Same | No change required. |
| **ASGS4** | Remoteness areas for NSW, VIC, TAS, ACT | 1- Major cities  2- Inner regional  3- Outer regional  4- Remote/ very remote | Same | Same | No change required. |
| **Mental_Health** | Mental health diagnosis | 0- Missing  1- Yes  2- No | Same | Same | No change required. |
| **Chronic** | Chronic pain diagnosis | 0- Missing  1- Yes  2- No | Same | Same | No change required. |

**Table S2. Variable coding and recategorisation**

| **NDSHS Label** | **Variable** | **Measure** | **Response options** | **Collapsed categories** | **Recoding** |
| --- | --- | --- | --- | --- | --- |
| CURF_Age | Age | What is your current age? (i.e. the age you turned at your last birthday) | Age in years | None - retain as continuous variable | None required |
| Gender (2022-23) | Gender | How do you describe your gender? | - Man or male - Woman or female - Non-binary - I use another term - Prefer not to answer | - Male - Female - Other / Missing | Recoded in supplied data   - Other / Missing: Non-binary OR I use another term OR Prefer not to answer OR Missing |
| Sex (2016, 2019) | Sex | What is your sex? | - Male - Female - Other | - Male - Female - Other / Missing | - Other / Missing: Non-binary OR I use another term OR Prefer not to answer OR Missing |
| MaritalStatus | Relationship status | Which one of the following best describes your present marital status? | - Never married - Divorced / separated / widowed - Married / defacto | - Partnered - Not partnered - Missing | - Partnered: Married / defacto - Not partnered: Never married OR Divorced / separated / widowed |
| NumberPpl | Household size | Derived variable: Number of people in household | Number of people | - Living alone - Not living alone - Missing | - Living alone: 1 - Not living alone: More than 1 |
| Employment2 | Employment status | Recoded variable from questions ZZ5a and ZZ5b | - Currently employed - Unemployed / looking for work - Not in labour force | - Employed - Not employed - Missing | - Employed: Currently employed - Not employed: Unemployed/Looking for work OR Not in labour force |
| EducationStatus | Education level | Recoded variable from questions ZZ9, ZZ11, and ZZ12 | - Year 11 or less (includes Certificate I or II) - Completed year 12 - Certificate III or IV - Diploma - Bachelor degree or higher | - High school or lower - Above high school - Missing | - High school or lower: Year 11 or less OR Completed Year 12 - Above high school: Certificate III or IV OR Diploma OR Bachelor degree or higher |
| PersIncome | Personal income | Which of the following groups would represent your personal annual income, before tax, from all sources? | - $156,000 or more ($3,000 or more/week) - $104,000 - $155,999 ($2,000 - $2,999/week) - $91,000 - $103,999 ($1,750 - $1,999/week) - $78,000 - $90,999 ($1,500 - $1,749/week) - $65,000 – $77,999 ($1,250 – $1,499/week) - $52,000 – $64,999 ($1,000 – $1,249/week) - $41,600 – $51,999 ($800 – $999/week) - $33,800 – $41,599 ($650 – $799/week) - $26,000 – $33,799 ($500 – $649/week) - $20,800 – $25,999 ($400 – $499/week) - $15,600 – $20,799 ($300 – $399/week) - $7,800 – $15,599 ($150 – $299/week - $1 – $7,799 ($1 – $149/week) - Nil or negative income - Prefer not to say - Don't know | - Higher - Lower - Missing | - Higher: $65,000+ - Lower: Below $65,000 including nil or negative   Note: median personal income in Australia is ~$55,000 and low-income tax offset is $65,000 |
| SEIFA | Socioeconomic status | Derived variable: The Index of Relative Socio-Economic Advantage and Disadvantage | - Lowest - 2 - 3 - 4 - Highest | - Advantaged - Disadvantaged - Missing | - Advantaged: 3 OR 4 OR 5 - Disadvantaged: 1 OR 2 |
| ASGS 3 (NSW, VIC, TAS, ACT) and ASGS4 (QLD, SA, WA, NT) | Geographic remoteness | Derived variable | - Major cities - Inner regional - Outer regional (ASGS4) - Outer regional, remote or very remote (ASGS3) - Remote or very remote (ASGS4) | - Major city - Regional, remote, or very remote - Missing | ASGS 3 used for NSW, VIC, TAS, ACT  ASGS4 used for QLD, SA, WA, NT |
| B1 | General health status | In general, would you say your health is …? | - Excellent - Very good - Good - Fair - Pair | - Good health - Poor health - Missing | - Good health: Excellent OR very good OR good - Poor health: Fair OR poor health |
| MentalHealth | Mental health diagnosis | Recoded variable from question B4 | - Yes - No | - Yes - No - Missing | Already recoded in supplied data |
| Chronic | Chronic pain diagnosis | Recoded variable from question B4 | - Yes - No | - Yes - No - Missing | Already recoded in supplied data |
| K10rank | Psychological distress | Recoded variable from questions B5-B15: The Kessler Psychological Distress Scale (K10) | - Low (10 - 15) - Moderate (16 - 21) - High (22 - 29) - Very High (30 - 50) | - Low/moderate - High/very high - Missing | - Low/moderate: low OR moderate - High/very high: high OR very high |
| EverInj | Lifetime non-medical injecting drug use | Derived variable: Have you ever injected any drugs apart from those prescribed to you? | - Yes - No | - Yes - No - Missing | Already recoded in supplied data |
| EverAnyIllicit | Lifetime illicit non-pharmaceutical drug use | Derived variable | - Yes - No | - Yes - No - Missing | Ever used marijuana/cannabis, ecstasy, methamphetamine/amphetamine, cocaine, inhalants, hallucinogens, heroin, ketamine, or GHB/GBL/1,4-BD |

**Table S3. Comparison of key sociodemographic and health characteristics between the NoNMU subsample and the remainder of the full NoNMU sample**

| **Characteristic** | **NoNMU subsample**  (n=1,602) | **NoNMU full sample**  (n=62,532) | ***p*** |
| --- | --- | --- | --- |
| Age, Mean | 44.5 | 45.4 | .164 |
| Male, % | 50.6 | 49.2 | .370 |
| Partnered, % | 59.7 | 60.2 | .777 |
| Living alone, % | 8.6 | 8.7 | .768 |
| Above high school education, % | 61.2 | 58.6 | .110 |
| Currently employed, % | 64.1 | 63.3 | .602 |
| Personal income ≥$65,000^§^, % | 37.0 | 37.4 | .800 |
| Disadvantaged socioeconomic status, % | 37.9 | 37.9 | .970 |
| Regional, remote, very remote area, % | 27.7 | 28.4 | .652 |
| Poor general health, % | 12.3 | 11.5 | .433 |
| High/very high distress, % | 14.4 | 14.3 | .939 |
| Chronic pain diagnosis, % | 9.8 | 10.1 | .745 |
| Mental health diagnosis, % | 16.3 | 16.3 | .996 |
| Lifetime illicit non-pharmaceutical drug use, % | 38.7 | 39.3 | .681 |
| Lifetime injecting drug use, % | 1.6 | 1.3 | .491 |

*Note*: Survey-weighted Wald and Rao–Scott F tests were used to assess differences between the NoNMU subsample and the remainder of the full sample. The full sample excludes individuals included in the NoNMU subsample to ensure independence of groups.

**Table S4. Completed pooling studies critical appraisal checklist**

The checklist comprises a total of 15 questions for one stage pooling strategies. Scores are categorised into three levels: less than 40% as low quality, 40-70% as intermediate, and higher than 70% as high quality.

Response options: Yes (1), No (0), Unclear (0), Not Applicable or NA (0)

| **Question** | **Item** | **Answer** | **Location** |
| --- | --- | --- | --- |
| 1 | Cleary Stated Which Databases to Be Pooled | 1 | Methods – Study design |
| 2 | Clearly Stated About Pooling Strategy | 1 | Methods – Pooling survey waves |
| 3 | Clearly Stated How the Variables Were Recoded | 1 | Methods – Data cleaning and harmonisation, Supp. 2 |
| 4 | Clearly Stated the Data Harmonization Process | 1 | Supp. 1 |
| 5 | Clearly Stated About a Quality Control Check After the Harmonization Process | 1 | Methods – Pooling survey waves |
| 6 | Utilization Of Survey Weight Variable | 1 | Methods – Survey weights |
| 7 | Utilization Of PSU or Cluster Variable | 1 | Methods – Multinomial logistic regression |
| 8 | Utilization Of Strata Variable | 1 | Methods – Multinomial logistic regression |
| 9 | Adding a Cycle Effect into the Model | 1 | Methods – Pooling survey waves |
| 10 | Dealing with Heterogeneity | 1 | Supp. 5 |
| 11 | Clearly Stated the Effect Used in the Analytical Model | 1 | Methods – Multinomial logistic regression |
| 12 | Study Used a Multilevel Regression | 0 | Used survey-weighted multinomial logistic regression |
| 13 | Study Dealing with Missing Data | 1 | Methods – Missing data |
| 14 | Clearly Stated About the SE/CI Estimation Method | 1 | Methods – Descriptive statistics and prevalence estimates |
| 15 | Study Performed Sensitivity Analysis | 1 | Supp. 7 |
| Total | Total ‘Yes’ (1) = 14  Total score = 14/15 = 93.3%  Quality category: High quality (> 70%) | | |

*Source*: Mayen et al. *BMC Medical Research Methodology* (2024) 24:279

**Table S5. Distribution of key variables by survey wave (total sample, weighted %)**

| **Variable** | **2016** | **2019** | **2022-23** | **Total** |
| --- | --- | --- | --- | --- |
| **Age, Mean** | 44.9 | 45.2 | 45.8 | 45.3 |
| **Sex/Gender, %** |  |  |  |  |
| Male | 49.3 | 49.2 | 48.7 | 49.1 |
| Female | 50.7 | 50.8 | 49.8 | 50.4 |
| Other / Missing | 0 | 0 | 1.5 | 0.5 |
| **Household size, %** |  |  |  |  |
| Living alone | 8.7 | 8.8 | 9.0 | 8.8 |
| Not living alone | 91.3 | 91.2 | 91.0 | 91.2 |
| Missing | 0 | 0 | 0 | 0 |
| **Socioeconomic status, %** |  |  |  |  |
| Advantaged | 59.5 | 61.1 | 65.3 | 62.0 |
| Disadvantaged | 40.5 | 38.9 | 34.7 | 38.0 |
| Missing | 0 | 0 | 0 | 0 |
| **General health status, %** |  |  |  |  |
| Good general health | 88.0 | 88.1 | 87.7 | 88.0 |
| Poor general health | 11.6 | 11.5 | 11.7 | 11.6 |
| Missing | 0.4 | 0.4 | 0.4 | 0.4 |
| **Psychological distress, %** |  |  |  |  |
| Low/moderate distress | 87.4 | 85.1 | 81.9 | 84.8 |
| High/very high distress | 12.0 | 14.3 | 17.4 | 14.6 |
| Missing | 0.6 | 0.6 | 0.7 | 0.6 |
| **Chronic pain diagnosis, %** |  |  |  |  |
| Yes | 9.5 | 10.1 | 9.7 | 9.8 |
| No | 85.1 | 84.8 | 86.6 | 85.4 |
| Missing | 5.4 | 5.0 | 3.9 | 4.8 |
| **Lifetime illicit drug use (non-pharmaceutical), %** |  |  |  |  |
| Yes | 36.6 | 38.4 | 42.6 | 39.2 |
| No | 62.4 | 60.4 | 54.8 | 59.2 |
| Missing | 1.0 | 1.1 | 2.6 | 1.6 |

**Sensitivity analyses for multinomial logistic regression**

**Table S6. Excluding missing cases**

| **Variable** | **NMGU** | | **NMOU** | |
| --- | --- | --- | --- | --- |
|  | **RRR (95% CI)** | ***p*** | **RRR (95% CI)** | ***p*** |
| Age (continuous) | 1.01 (0.98–1.03) | .592 | 1.00 (0.99–1.01) | .717 |
| Female Sex/Gender | 0.79 (0.42–1.47) | .454 | 0.91 (0.73–1.13) | .378 |
| Chronic pain diagnosis | 5.48 (3.05–9.83) | <.001 | 1.93 (1.44–2.59) | <.001 |
| High/very high psychological distress | 4.08 (2.11–7.87) | <.001 | 2.30 (1.76–3.01) | <.001 |
| Disadvantaged socioeconomic status | 3.00 (1.60–5.64) | .001 | 1.23 (0.98–1.53) | .072 |
| Lifetime illicit non-pharmaceutical drug use | 2.54 (1.11–5.84) | .028 | 3.61 (2.88–4.52) | <.001 |
| 2019 survey wave | 1.84 (0.76–4.44) | .177 | 1.01 (0.78–1.31) | .910 |
| 2022-23 survey wave | 2.23 (0.89–5.57) | .087 | 0.77 (0.59–1.00) | .053 |

**Table S7. Using original unadjusted survey weight**

| **Variable** | **NMGU** | | **NMOU** | |
| --- | --- | --- | --- | --- |
|  | **RRR (95% CI)** | ***p*** | **RRR (95% CI)** | ***p*** |
| Age (continuous) | 1.01 (0.99–1.03) | .408 | 1.00 (1.00–1.01) | .303 |
| Female Sex/Gender | 1.00 (0.55–1.82) | .989 | 0.90 (0.73–1.09) | .282 |
| Chronic pain diagnosis | 5.69 (3.19–10.15) | <.001 | 1.83 (1.37–2.45) | <.001 |
| High/very high psychological distress | 4.33 (2.35–7.96) | <.001 | 2.39 (1.84–3.11) | <.001 |
| Disadvantaged socioeconomic status | 3.42 (1.88–6.21) | <.001 | 1.22 (0.98–1.50) | .069 |
| Lifetime illicit non-pharmaceutical drug use | 2.99 (1.31–6.84) | .010 | 3.50 (2.82–4.36) | <.001 |
| 2019 survey wave | 2.01 (0.87–4.66) | .103 | 1.01 (0.80–1.29) | .905 |
| 2022-23 survey wave | 2.37 (1.00–5.61) | .051 | 0.75 (0.58–0.97) | .026 |

**Figure S1. Directed acyclic graph and covariate adjustment strategy**

To inform covariate selection, we developed a directed acyclic graph (DAG) representing hypothesised relationships between psychological distress, non-medical use of pain medications (NMU), and a set of sociodemographic and health variables (below figure). The DAG was developed collaboratively by three members of the research team (AM, TX, SN) with expertise in pharmacy, psychology, public health, and statistics. It was created in DAGitty (<https://www.dagitty.net/>) and reflects our assumptions based on prior evidence.


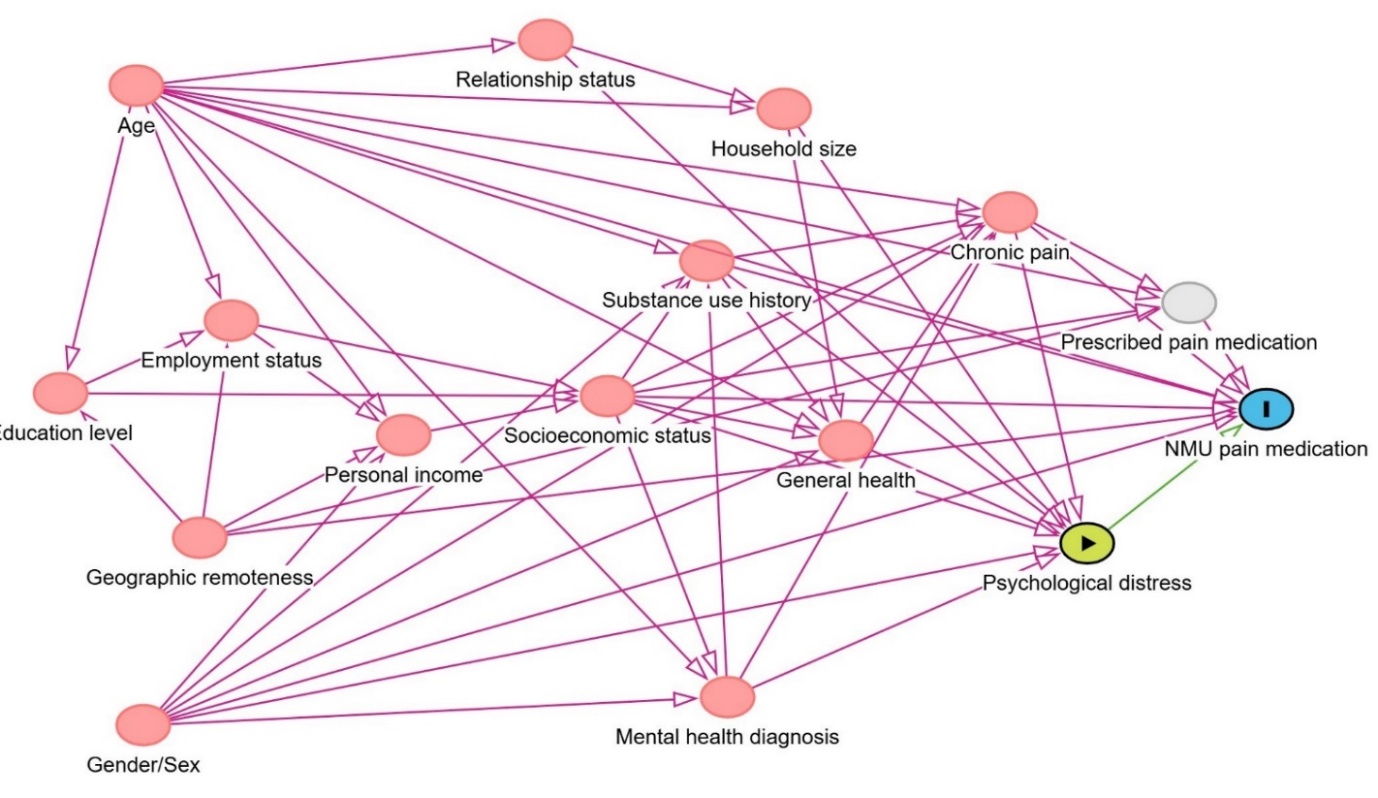


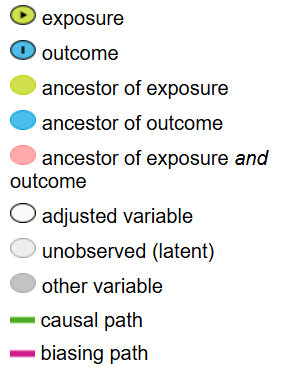


Using DAGitty, we identified a minimal sufficient adjustment set to estimate the total effect of psychological distress on NMU pain medication use: Age, chronic pain, gender/sex, socioeconomic status, substance use history (i.e., lifetime illicit non-pharmaceutical drug use)

This set ensures confounding control while avoiding unnecessary adjustment that could reduce precision or introduce bias.
